# Immunization Combined with Ferroptosis Related Genes to Construct a New Prognostic Model for Head and Neck Squamous Cell Carcinoma

**DOI:** 10.3390/cancers14174099

**Published:** 2022-08-24

**Authors:** Linhui Yang, Zhiwei Chen, Yunliang Liu, Xiaoyan Wang, Jing Li, Qing Ye

**Affiliations:** 1 Department of Otolaryngology—Head and Neck Surgery, Fujian Provincial Hospital, Shengli Clinical Medical College of Fujian Medical University, Fuzhou 350001, China; 2 The Graduate School, Fujian Medical University, Fuzhou 350001, China; 3 Fujian Provincial Maternity and Children’s Hospital, Fuzhou 350001, China

**Keywords:** immune-ferroptosis, head and neck squamous cell carcinoma, prognosis, biomarkers, immune response

## Abstract

**Simple Summary:**

Immunity combined with ferroptosis is being considered as a new tumor treatment modality, and its regulation in head and neck squamous cell carcinoma is still unknown. The purpose of this study was to look into the potential molecular biological roles of immune ferroptosis genes in head and neck squamous cell carcinoma. The 12-IFRM signatures were successfully constructed and classified into high- and low-risk groups using the TCGA database and related data resources. In patients with head and neck squamous cell carcinoma, feature-based risk scores were more predictive of survival than traditional clinicopathological features. Furthermore, the expression of CD8^+^T cells and macrophage M0 differed significantly between the two groups. The expression of TNFSF9 and CD44 in the high-risk groups was significantly increased compared with the low-risk groups. Next, we found a higher proportion of high-risk mutations than in the low-risk group. In addition, the high-risk group was more sensitive to some chemotherapy drugs. Finally, we performed correlation analysis on the model genes. In this paper, the 12-IFRM signatures was developed with promising application prospects for predicting the clinical outcomes and treatment outcomes in head and neck squamous cell carcinoma.

**Abstract:**

Ferroptosis is a new type of programmed cell death that plays a pivotal role in a variety of tumors. Moreover, immunity is closely related to ferroptosis. However, immune-ferroptosis-related mRNAs (IFRMs) are still not fully understood in the regulation of head and neck squamous cell carcinoma (HNSC). The purpose of this paper was to investigate the IFRMs prediction of HNSC and its possible molecular biological role. RNA-Seq and related clinical data were mined from the TCGA database, ImmPort database, GeneCards database, FerrDb database, and previous data. In R software, the “DESeq2” package was used to analyze the differential expression of IFRMs. We used univariate Cox analysis to judge the prognosis of the IFRMs. Using the least absolute shrinkage and selection operator (LASSO) and Cox regression, a prediction model for 12 IFRMs was established. In this study, the Kaplan–Meier survival curve and receiver operating characteristic (ROC) curve analysis were used to evaluate the prediction results. Moreover, factors such as immune landscape, somatic mutations, and drug susceptibility are also discussed. We successfully constructed the signature of 12-IFRMs. The two risk groups were classified according to the risk score obtained by this signature. Compared with conventional clinicopathological features, the characteristic-based risk score was more predictive of survival in patients with HNSC. Furthermore, the expression of CD8^+^T cells and macrophage M0 differed significantly between the two groups. Moreover, the expression of TNFSF9 and CD44 in high-risk groups was significantly increased compared with the low-risk groups. Then, we found a higher proportion of high-risk mutations than in the low-risk group. Next, the high-risk group was more sensitive to chemotherapy drugs such as bosutinib, docetaxel, erlotinib, gefitinib, imatinib, lapatinib, and sorafenib. Finally, an in-depth analysis of the association and potential value of the 12 genes was performed. In summary, the 12-IFRM signatures established in this paper had good application prospects and could be effectively used to predict the clinical outcome and treatment response of head and neck squamous cell carcinoma.

## 1. Introduction

Head and neck squamous cell carcinoma (HNSC) is a malignant disease of the head and neck with a high incidence and high fatality rate [1]. In recent years, the incidence and mortality of the disease have been increasing. Even with the continuous development of tumor screening and treatment technology, the current therapy is still dominated by surgery, chemotherapy, and radiation therapy [2]. However, there is currently no effective treatment for head and neck squamous cell carcinoma [3]. At the same time, with the continuous improvement in medical technology and other aspects, it is also very necessary to explore the tumor markers and potential treatment targets.

In 2012, researchers found that erastin promotes cell death, mainly characterized by increased double membrane density, iron-dependent lipid peroxidation, regulated by the cystine transport pathway, and mitochondrial contraction, which was first named ferroptosis [4]. Ferroptosis is a lipid peroxidation and iron-dependent cell death due to decreased activity of glutathione peroxidase 4 (GPX4) and the accumulation of lipid reactive oxygen species (ROS) [5]. Now, it has been found that inducing ferroptosis has become a means of eliminating tumor cells, especially for resistant tumors [6]. Ferroptosis can act as a tumor suppressor by eliminating damaged cells [7] and has been reported in various cancer types [8,9,10,11,12]. It has been reported that capsaicin induces ferroptosis by inactivating the SLC7A11/GPX4 signaling pathway [13]. Free docosahexaenoic acid can effectively promote ferroptosis by increasing intracellular lipid peroxidation [14]. A study found that Hedyotis diffusa injection induced ferroptosis in lung adenocarcinoma cells by inhibiting Bcl2 and promoting the regulation of VDAC2/3 by Bax [15]. Epithelial membrane protein 1 (EMP1) deficiency promotes bladder cancer cell migration and confers resistance to ferroptosis/oxidative stress, thereby promoting bladder cancer metastasis via PPARG [16]. Another study showed that EMP1 overexpression could promote gefitinib resistance by targeting the MAPK pathway, which might be a therapeutic target for HNSC [17]. Circular RNA circACAP2 suppresses ferroptosis in cervical cancer during malignant progression through miR-193a-5p/GPX4 [18]. It was also found that targeting ferroptosis might help suppress cancer metastasis [19]. Additionally, numerous experiments have shown that ferrptosis and immunity could play a role in many tumors [20,21,22]. For example, it has been reported that CD8^+^T lymphocytes play a regulatory role in tumor immunotherapy [22]; therefore, iron oxide-loaded nanovaccines (IONV) could improve the immune efficacy by combining ferroptosis with immunity [23]. Therefore, the regulation of ferroptosis and immunity has now been regarded as a new tumor treatment target [22,24,25]. In head and neck squamous cell carcinoma, few therapeutic targets related to immune combined ferroptosis have been found. Therefore, in the diagnosis and treatment of HNSC, further screening based on immune-ferroptosis-related genes (IFRGs) and clinical samples is necessary.

In this study, we conducted a deep mining of HNSCs in the TCGA database. In Section 1, we performed differential analysis of RNA-Seq data from HNSCs. The differentially expressed genes were crossed with immune ferroptosis genes to obtain immune ferroptosis differentially expressed genes (IFRDEGs), and then prognostic analysis was performed to obtain PIFRDEGs. In Section 2, using the LASSO-Cox algorithm, we built a risk prediction model based on 12 genes from PIFRDEGs. The model was subsequently validated satisfactorily. In Section 3, we use a heatmap to further illustrate the expression of 12 genes in the risk model. In parallel, we performed a differential analysis of the risk model in Section 4 and a functional enrichment analysis of the differential genes. Next, Section 5 examines the immune infiltration and immunological checkpoints to investigate the influence of high- and low-risk on immunity. At the same time, the relationship between risk model and tumor mutation is evaluated in Section 6. We examine the relationship between high- and low-risk and drug susceptibility in Section 7. Finally, a deeper analysis of the connections and potential usefulness among these 12 genes is conducted. In conclusion, we delved into the relationship between IFRGs and HNSCs. This lays a foundation for clinical diagnosis and treatment, and provides a new direction for therapeutic targets.

## 2. Materials and Methods

### 2.1. Data Collection

We collected RNA-Seq data (https://portal.gdc.cancer.gov/projects/TCGA) (accessed on 16 June 2022) from 543 HNSC samples from TCGA including 44 normal samples and 499 tumor samples with corresponding HNSC clinical data. These RNA-Seq data, which also included mRNA and lncRNA, were collected. The fragments per kilobase of transcript per million mapped read (FPKM) calculations were calculated by normalizing reads by dividing it by the gene length and the number of encoded gene reads mapped to the protein. The perl approach was used to convert Ensembl IDs to official gene symbols, which were then processed by log2. Tumor mutation burden (TMB) usually refers to the number of somatic non-synonymous mutations or all mutations per megabase in the gene region detected by whole-exome sequencing or targeted sequencing in the tumor samples, that is, somatic gene coding errors, the number of base substitution, gene insertion, or deletion errors. The “MAF” package was used to test the number of somatic nonsense point mutations in each sample. These further shed light on how HNSC drives genetic somatic changes in low-risk and high-risk samples.

### 2.2. Identification of Immune-Ferroptosis-Related mRNA

The IFRM sequences of Homo sapiens were downloaded from the ImmPort database (www.immport.org/home) (accessed on 16 June 2022), GeneCards database (www.genecards.org/) (accessed on 16 June 2022), FerrDb database (http://www.zhounan.org/ferrdb/) (accessed on 16 June 2022), and previous documents [26,27]. In this study, a total of 17,500 immune-related genes and 398 ferroptosis-related genes were obtained.

### 2.3. Differential Expression Analysis

We incorporated the normalized matrix data into the R software, and “DESeq2” [28] was used to compare the mRNA expression matrix of HNSC with the normal samples. Thus, differentially expressed mRNAs (DEmRNAs) were obtained. The criteria for DEmRNA were |log (2) (fold change)| > 1 and P. adj < 0.05 [29].

### 2.4. Construction of Immune-Ferroptosis-Related Prognostic Signature

The intersection genes between IFRGs and DEmRNAs were screened by the “survival” package of Cox univariate analysis. A total of 499 patients were divided into two groups at a 1:1 ratio. On this basis, regression was performed using the least absolute shrinkage and selection operator (LASSO)-Cox method. Finally, 12 optimized prognostic models were constructed according to the selection of the optimal penalty parameter λ associated with the minimum 10× cross-validation. The risk score formula for each patient’s immune-ferroptosis-related to prognosis was as follows: risk score = Ʃ(Expi × βi). Among these indicators, βi represents the Cox risk ratio coefficient of each factor, and Expi represents the expression of the gene. Based on this, the subjects were assessed for risk and divided into high-risk groups and low-risk groups. The Kaplan–Meier curve uses the “survminer” package and Cox test to compare the overall survival (OS) of high- and low-risk groups. The “timeROC” package was used to generate a receiver operating characteristic curve (ROC) [30] to assess the predictive accuracy of the signature. To test the model’s viability, a risk score was computed in the validation and general cohorts using the same methodology as in the training cohort, and then the validation procedure described above was used.

### 2.5. Gene Set Enrichment Analysis (GSEA)

We used the “limma” [31] packet to identify differentially expressed genes across the high-risk and low-risk groups (|log (2) (fold change)| > 1 and FDR < 0.05). For reference, the’ c2. cp. v7. 2. symbols. gmt ‘genome was obtained from the molecular signature database (MSigDB) (http://software.broadinstitute.org/gsea/msigdb) (accessed on 16 June 2022) [32]. We performed GSEA analysis with at least 1000 alignment tests per analysis [33] to find the significant differences between the high-risk and low-risk groups, *p* < 0.05, FDR < 0.25. From this, we were able to find the main major biological pathways.

### 2.6. Functional Enrichment Analysis

Gene Ontology (GO) is a database widely used in bioinformatics, in which seven million gene annotations are stored, about one tenth of which have been experimentally proven [34]. GO analysis is based on candidate genes to analyze the cellular component (CC), biological process (BP), and molecular function (MF). The Kyoto Encyclopedia of Genes and Genomes (KEGG) is a database for the analysis of advanced functional and biological systems through genome sequencing and high-throughput experimental techniques generated from large molecular datasets [35]. The KEGG generates a pathway map based on the relationship network formed by gene sequence, genome information, metabolism, disease, etc. In order to analyze the enrichment of gene biological functions and pathways, we performed enrichment analysis through the “clusterProfiler” package [36], and performed ID conversion through the “org. Hs. eg. db” package, and the “ggplot2” package was used for data visualization. The filter condition for *p* < 0.05 was set. Moreover, GO and KEGG analysis were also performed on the subsequent model-related genes.

### 2.7. Immune Cell Infiltration and Immune Microenvironment Evaluation

The ESTIMATE algorithm [37] is a method to assess immune cell infiltration and the tumor microenvironment based on gene expression, which can calculate the immune scores, stromal scores, and estimated scores between samples. CIBERSORT [38] is a method that uses expression data to reflect the cellular composition of complex tissues by quantitatively measuring the abundance of 22 tumor-infiltrating immune cell types in a sample. CIBERSORT’s LM22 specifies 22 immune cell subpopulations from the CIBERSORT database (http://CIBERSORT.stanford.edu/) (accessed on 16 June 2022). The correlations between the risk scores and tumor-infiltrating immune cells can be assessed. Immune checkpoint-related gene expression levels may be connected to therapeutic responsiveness to immune checkpoint inhibitors. The correlation between risk scores and immune checkpoints was explored by analyzing the gene expression between the high-risk groups and low-risk groups.

### 2.8. Model Gene Alteration Analysis

To explore the genetic alterations of model genes in HNSC, we further analyzed them through the cBioPortal [39] database (https://www.cbioportal.org/) (accessed on 16 June 2022). The cBioPortal is a friendly tool resource that provides gene mutation, copy number variation, and transcriptional expression data from samples of various cancer subtypes. We selected “TCGA-HNSC data” in the “query” module, entered 12 model genes and queried the gene alteration situation. In the “Summary of Cancer Types” module, all HNSC tumors were for observed alteration frequency, mutation type, and result of copy number alteration. The mutation site information of *CDKN2A* can be displayed in the protein three-dimensional structure schematic diagram through the “Mutations” module.

### 2.9. Prediction of Drug Sensitivity

The therapeutic response to known chemotherapeutic agents (bosutinib, docetaxel, erlotinib, gefitinib, imatinib, lapatinib, lenalidomide, metformin, methotrexate, nilotinib, rapamycin, sorafenib) was estimated using the “pRRophetic” [40] package. The content of each chemotherapeutic agent was calculated in the HNSC specimens by constructing a ridge regression model based on the Genomics of Drug Sensitivity in Cancer (GDSC) database (www.cancerRxgene.org) (accessed on 16 June 2022) [41] and transcriptome data to obtain the half-maximal inhibitory concentration (IC50).

### 2.10. Construction of Gene-Gene and Gene-Protein Networks

The GeneMANIA [42] (https://genemania.org/) (accessed on 16 June 2022) is a database for analyzing gene interactions or shared functions by predicting gene functions. Model genes established a gene–gene interaction network through GeneMANIA. The STRING [43] database (https://string-db.org/) (accessed on 16 June 2022) is a search tool for analyzing the gene and protein interaction relationship. This provides users with easy access to unique, wide-ranging experiments, and predicted interaction information. Model genes construct protein–protein interaction (PPI) networks through STRING.

### 2.11. Statistical Analysis

**Figure 1:**
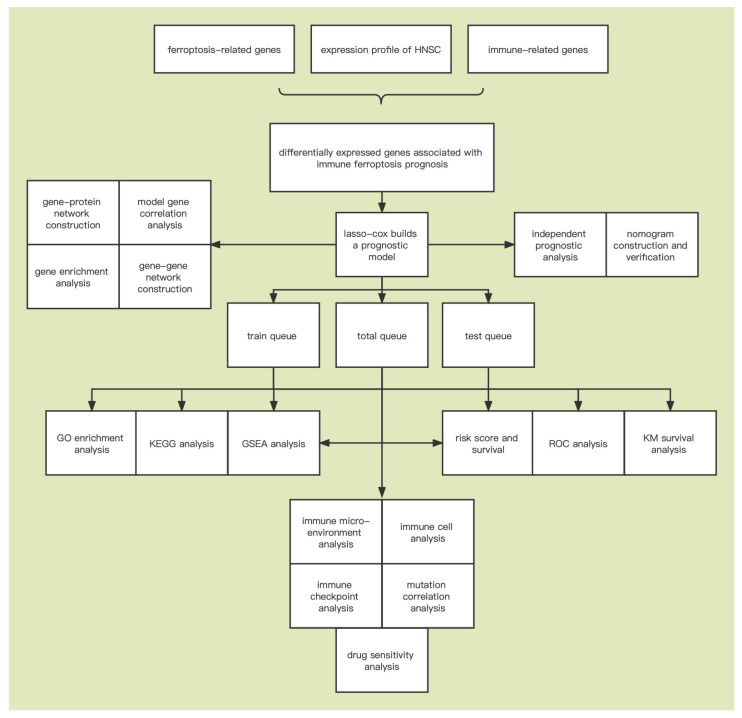
The flow chart of the data collection and analysis.

All statistical analyses were performed by using R software (version 4.1.2) using the Cox univariate and multivariate regression analyses to identify independent prognostic factors for overall survival. Survival analysis was performed using Cox univariate regression analysis. Time-dependent ROC curve analysis was used to measure the degree to which overall survival prognostic models predicted outcomes. Differences in the proportions of clinical features were analyzed by the chi-square test. Correlation analysis of IFRMs was performed using Spearman’s correlation method. Tumor immune infiltrating cells were compared using the Wilcox test. Visualization was conducted using the “ggplot2”, “pheatmap”, and “forestplot” packages. *p* < 0.05 was used to determine the statistical significance. The overall flow chart is shown in Figure 1.

## 3. Results

### 3.1. Identification of Immune-Ferroptosis-Related Differentially Expressed mRNAs in HNSC

**Figure 2:**
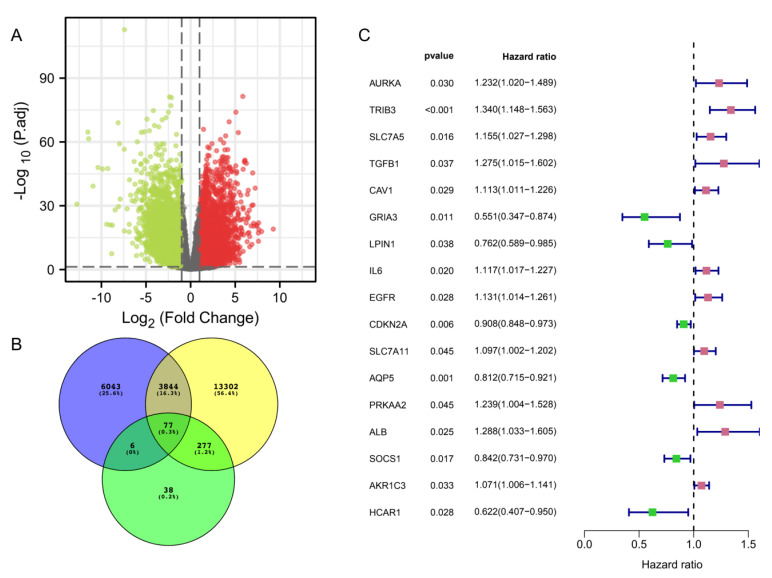
Screening of the differential mRNAs in immune ferroptosis with prognosis. (**A**) Differential gene volcanogram. Red is the upregulated genes and green is the downregulated genes. (**B**) Venn diagram identifying the differentially expressed mRNA and immune-ferroptosis-related mRNA. Blue are differentially expressed genes; yellow are immune-related genes; green are ferroptosis-related genes. (**C**) Prognostic forest diagram of the differentially expressed immune-ferroptosis-related mRNA.

We obtained 543 HNSC data from the TCGA database (https://portal.gdc.cancer.gov/repository) (accessed on 16 June 2022). By comparing the TCGA-HNSC samples with normal tissues, we found 9970 differentially expressed mRNAs (DEmRNAs) (log (2) |FC| > 1, *p* adj < 0.05), of which 6166 were upregulated and 3804 were downregulated (Figure 2A).

Subsequently, we obtained 17,500 immune-related genes through the ImmPort database, GeneCards database, and 398 ferroptosis-related genes through the FerrDb database and previous studies [26,27], and then obtained 77 genes associated with differentially expressed genes, immune-related genes, and ferroptosis-related genes by intersection (Figure 2B and Supplementary Table S1). The prognostic potential of IFRDEGs was analyzed by Cox univariate regression using OS data from the HNSC patients in the TCGA database. Finally, we found 17 prognostic IFRDEGs (PIFRDEGs) in HNSC (Figure 2C). Eleven of the PIFRDEGs were “risky” and six were “protected”.

### 3.2. IFRMs Prognostic Model Construction and Validation

According to the TCGA-HNSC database, we randomly divided the sample into two groups according to the method of 1:1, obtaining 250 people in the training group (125 people at high risk and 125 people at low risk), and 249 people in the validation group (107 people at high risk and 142 people at low risk).

**Figure 3:**
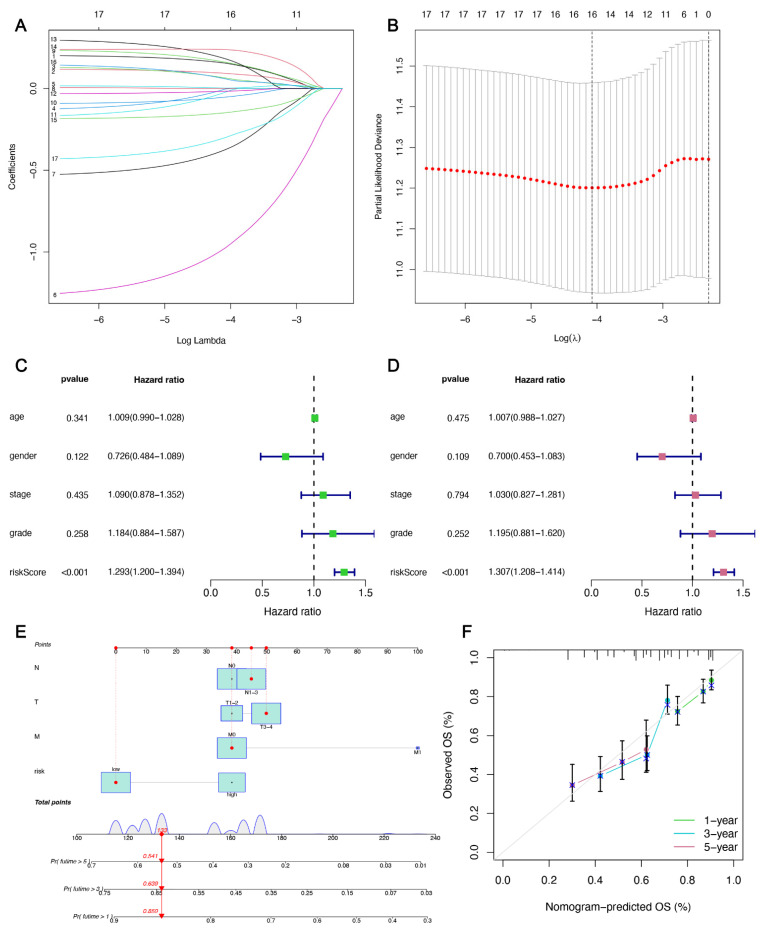
Constructing a prognostic model and analyzing the independent prognostic potential. (**A**,**B**) Utilizing minimal criteria, Cvfit, and lambda curves displaying LASSO regression were generated. (**C**,**D**) Results of the univariate Cox analysis and multivariate Cox analysis of OS characterized by 12-immune-ferroptosis-related mRNAs. (**E**) Nomogram predicting the 1-, 3-, and 5-year overall survival in HNSC patients. (**F**) The calibration curve used to evaluate the accuracy of the nomogram. The gray diagonal dashed line represents the ideal nomogram.

In the training group, LASSO regression analysis was performed on the genes obtained in the univariate COX regression analysis. Figure 3A shows a plot of the LASSO coefficients for 17 prognostic genes; Figure 3B is a ten-fold cross-validation plot used to adjust parameters in the LASSO model. Partial likelihood deviations were plotted against log (λ), where λ is the tuning parameter. The partial likelihood deviation values are shown in the graph, and the error bars represent the SE (standard deviation). Vertical dashed lines were drawn at the minimum standard and the 1–SE optimum. Here, we chose λ = 16, and these 16 prognostic genes were all included in the subsequent analysis. Subsequently, a prediction model consisting of 12 genes was obtained after multivariate regression analysis. In this model, each HNSC patient was given a risk score in the TCGA database according to the following formula: risk score = (0.259 × *AURKA* expression) + (0.154 × *SLC7A5* expression) + (−1.297 × *GRIA3* expression) + (−0.536 × *LPIN1* expression) + (0.228 × *EGFR* expression) + (−0.095 × *CDKN2A* expression) + (−0.160 × *SLC7A11* expression) + (0.308 × *PRKAA2* expression) + (0.270 × *ALB* expression) + (−0.208 × *SOCS1* expression) + (0.155 × *AKR1C3* expression) + (−0.457 × *HCAR1* expression) (Supplementary Table S2). (*AURKA*: *Aurora Kinase A*; *SLC7A5*: *Solute Carrier Family 7 Member 5*; *GRIA3*: *Glutamate Ionotropic Receptor AMPA Type Subunit 3*; *LPIN1*: *Lipin 1*; *EGFR*: *Epidermal Growth Factor Receptor*; *CDKN2A*: *Cyclin Dependent Kinase Inhibitor 2A*; *SLC7A11*: *Solute Carrier Family 7 Member 11*; *PRKAA2*: *Protein Kinase AMP-Activated Catalytic Subunit Alpha 2*; *ALB*: *Albumin*; *SOCS1*: *Suppressor Of Cytokine Signaling 1*; *AKR1C3*: *Aldo-Keto Reductase Family 1 Member C3*; *HCAR1*: *Hydroxycarboxylic Acid Receptor 1*).

To further assess the independent predictive power of risk models, we performed univariate Cox and multivariate Cox regression assessments based on the risk scores and associated clinical variables (age, gender, stage, grade). It was found that in the HNSC cohort, the risk score was associated with OS (*p* < 0.001) (Figure 3C). Furthermore, multivariate Cox regression resulted from the TCGA database indicated that only the risk distribution of these 12-IFRMs could serve as an independent prognostic factor for predicting the OS rates in the HNSC patients (*p* < 0.001) (Figure 3D).

A nomogram was established at the TCGA to predict the probability of survival in the HNSC patients. Each factor was assigned a score based on the T, N, M, and risk scores, and an overall nomogram score was obtained from the sum of the individual scores for all predictors. The 1-, 3-, and 5-year survival rates of patients were estimated from the predicted total score. As shown in Figure 3E, nomograms for 1, 3, and 5 years were constructed based on the predictive model (risk score) and clinical factors (T, N, M). To verify the accuracy of the model, we constructed calibration plots that showed that the 1-, 3-, and 5-year forecasts were close to ideal and also showed that the model had certain accuracy (Figure 3F).

**Figure 4:**
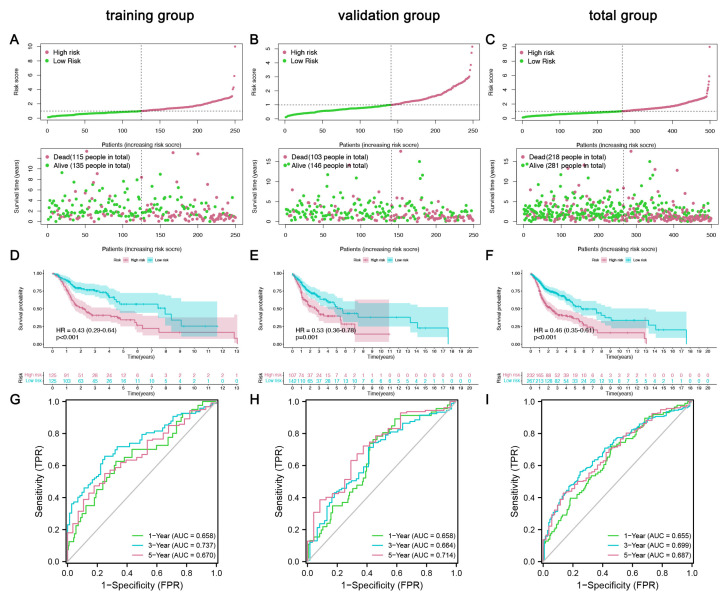
The mRNA signature model constructed and validated in the cohort. (**A**–**C**) Distribution of overall survival status and risk scores in the training, validation, and total groups. (**D**–**F**) Kaplan–Meier curves of survival status and survival time in the training, validation, and total groups. (**G**–**I**) ROC curves show the potential of prognostic immune-ferroptosis-related mRNA signatures in predicting 1-year, 3-year, and 5-year overall survival (OS) in the training, validation, and overall groups.

Afterward, we evaluated this 12-IFRM model. On this basis, subjects were divided into high-risk groups and low-risk groups according to the average level of risk scores. By visualizing the risk score and OS status, the results showed that in these two risk groups, the risk grouping was more reasonable (Figure 4A). A subsequent study using the Kaplan–Meier survival method showed that patients with HNSC in the low-risk group had a higher OS rate than those in the high-risk group (Figure 4D). Meanwhile, in this model, time-dependent ROC curves were also calculated. Areas under the curve (AUC) were maintained above 0.65 over 1, 3, and 5 years (Figure 4G). To verify the predictive power of this 12 mRNA trait, the profile, Kaplan–Meier survival curve, and time-dependent ROC curves were analyzed in the validation and the total group. Results in the validation group (Figure 4B,E,H) and the total group (Figure 4C,F,I) showed the same trend as the training group. Clearly, mortality was higher in the high-risk groups than in the low-risk groups.

### 3.3. Relationship between Risk Grouping and Clinicopathological Features

**Figure 5:**
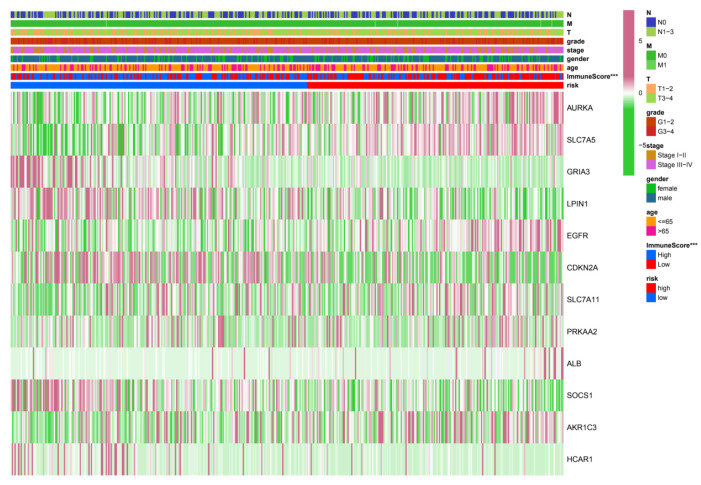
Heatmaps of the correlations between prognostic signatures and different clinicopathological features in the TCGA cohort. We found that these six genes (*PRKAA2*, *ALB*, *AURKA*, *EGFR*, *AKR1C3*, *SLC7A5*) tended to cluster more significantly with increasing risk. Conversely, the other six genes (*CDKN2A, SLC7A11*, *SOCS1*, *HCAR1*, *LPIN1*, *GRIA3*) tended to cluster more significantly with decreasing risk. (*** *p* < 0.001. ns—no significance.).

To further explore whether risk groups differed in gene expression and associated clinical variables, we performed heatmap visualizations. In the risk grouping of TCGA-HNSC, six mRNAs were considered risk mRNAs and the remaining six were protective mRNAs (Figure 5). We compared the clinicopathological features of the two risk subgroups. Interestingly, the immune scores (*p* < 0.001) of the two groups differed widely. It is suggested that this 12-mRNA signature has a significant potential to predict immunity in HNSC patients by assessing their risk scores by the relevant gene expression levels.

### 3.4. GSEA, GO, and KEGG Analysis Reveals Molecular Functions and Pathways

**Figure 6:**
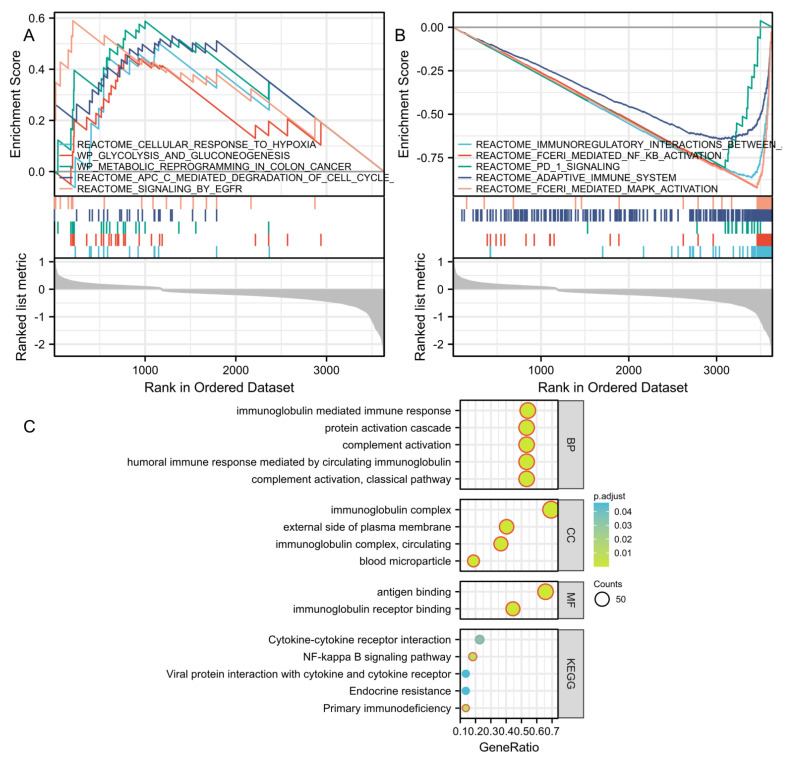
Analysis of the biological function and pathway enrichment in the high-risk and low-risk groups based on the prognostic characteristics of immune-ferroptosis-related mRNA. (**A**) The GSEA of the patients in the high-risk group. (**B**) The GSEA of patients in the low-risk group. (**C**) Bubble plot of the GO and KEGG functional pathway enrichment analysis. The size of the bubble is consistent with the number of count in the corresponding result record, which is the total number of intersections between the gene set and the molecules in the corresponding ID entry; the depth of the bubble color is consistent with the P. adj in the corresponding result record, which is the size of the *p* value after statistical test correction. The abscissa is the molecular ratio, which is consistent with the GeneRatio data in the corresponding analysis table. BP—biological process; CC—cellular component; MF—cellular component.

We further explored the relationship between biological processes and signaling pathways in risk groups classified according to the 12-IFRM signatures. Therefore, we performed a differentially expressed gene (DEG) analysis between the two risk groups. The DEG between the high-risk group and the low-risk group was determined by log (2) |FC| > 1 and FDR < 0.05, and GSEA was carried out. The results showed that many cancer-related pathways were enriched in high-risk groups such as cellular response to hypoxia, glycolysis and gluconeogenesis, metabolic reprogramming in colon cancer, APC mediated degradation of cell cycle proteins, signaling by EGFR (Figure 6A). In addition, many tumor immune pathways were enriched in the low-risk group such as immunoregulatory interactions between a lymphoid and a nonlymphoid cell, fceri mediated NF-KB, PD_1 signaling, adaptive immune system, and fceri mediated MAPK (Figure 6B).

To predict the functional enrichment information of high- and low-risk differential genes, we performed GO and KEGG enrichment analysis. KEGG analysis showed significant enrichment of many related pathways including primary immunodeficiency, NF-KB signaling pathway, cytokine–cytokine receptor interaction, endocrine resistance, and viral protein interaction with cytokine and cytokine receptor, similar to the results of GSEA (Figure 6C). GO analysis indicated the enrichment of biological processes (BP), molecular functions (MF), and cellular components (CC). Taken together, these results suggest that the risk score of the 12-mRNA signatures was primarily associated with immunity and biometabolism. Details of the GO and KEGG results are listed in Table 1.

### 3.5. Immune-Related Analysis of HNSC Patients Using the Prognostic Signature

**Figure 7:**
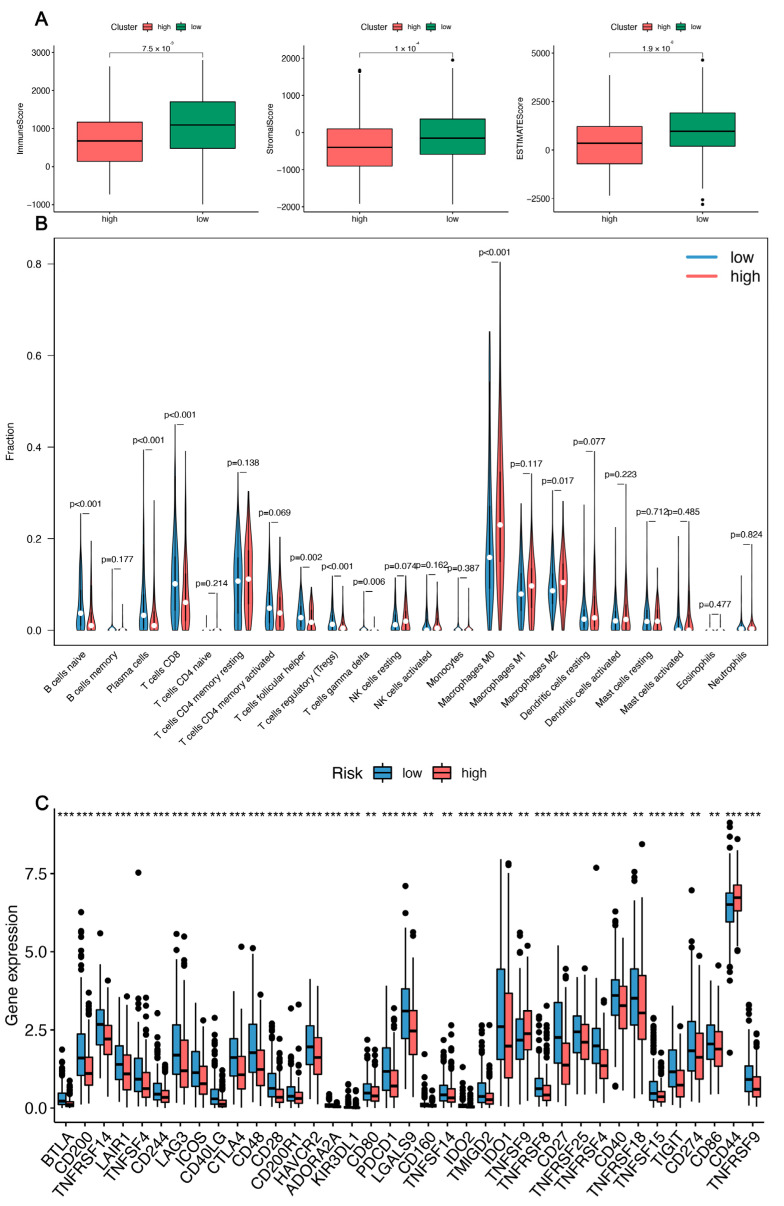
The analysis of different risk groups and immune-related conditions for HNSC. (**A**) The relationship between immune microenvironment and risk score was analyzed. The immune scores, stromal scores, and estimated scores were analyzed by the ESTIMATE algorithm. (**B**) Analysis of the immune infiltrating cells in high- and low-risk groups was performed by the CIBERSORT algorithm. (**C**) Boxplots of immune checkpoint expression and risk grouping. (** *p* < 0.01, and *** *p* < 0.001, ns—no significance.).

The tumor microenvironment, as the name implies, is the living environment in which tumor cells proliferate and metastasize in deep tissues. It consists of tumor cells, immune cells, stromal cells, and various active molecules, all of which play a role in tumor progression. In this study, HNSC patients were immunologically tested using CIBERSORT and ESTIMATE to explore their association with 12-IFRMs. In order to clarify the differences in immune cells in the risk groups, the stromal scores, immune scores, and estimate scores in the risk groups were compared in groups, and it was found that the high-risk group had significantly lower scores in these groups (*p* < 0.001) (Figure 7A). In addition, the immune cells in high-risk groups and low-risk groups were compared, and the results showed that naive B cells, plasma cells, CD8^+^T cells, T follicular helper cells, T cells regulatory (Tregs), T cells gamma delta, macrophage M0, and macrophage M2 were significantly different between different groups (*p* < 0.05) (Figure 7B).

We also compared the immune checkpoint expression levels in the risk groups. In Figure 7C, there were significant differences in genes at 37 checkpoints between the two groups. The expression of TNFSF9 and CD44 in the high-risk groups was significantly increased compared with the low-risk groups. These findings imply that the high-risk group’s immune microenvironment may be reduced by upregulating immunosuppressive cytokines and immune checkpoints.

### 3.6. Gene Mutation Analysis in the Model

**Figure 8:**
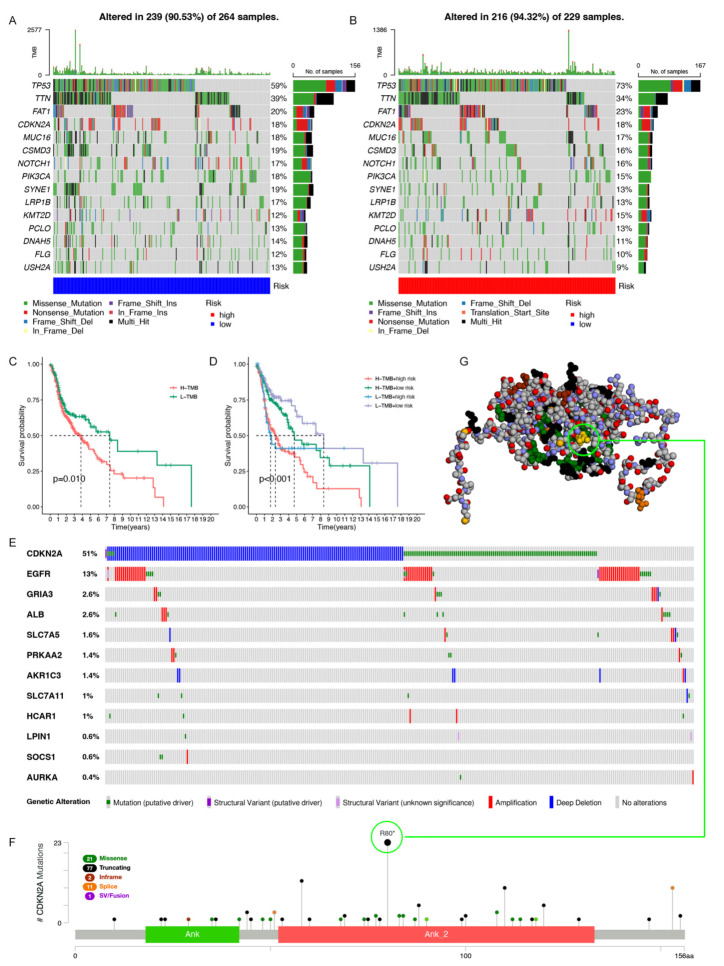
The gene mutation analysis. (**A**) Low-risk score oncoPrint map. (**B**) High-risk score oncoPrint map. (**C**) The Kaplan–Meier curves for high and low TMB groups. (**D**) The Kaplan–Meier curves for patients stratified by TMB and risk score. (**E**) A summary of the model genes’ structural variant, mutations, and copy-number alterations. (**F**) The mutation types, number, and sites of the *CDKN2A* genetic alterations. R80* is the most frequently altered mutation site in *CDKN2A*. (**G**) Three-dimensional structure of *CDKN2A*. The yellow structure is where R80* is located.

Several recent reports have pointed out that high tumor mutation burden (TMB) are significantly associated with the abundance of CD8^+^T cells, and can recognize tumor cells and predict immune status [44,45,46]. Therefore, we believe that TMB can be a predictor that cannot be underestimated. By exploring the correlation between TMB and risk score, we found the relationship between the genetic variation in the risk score subtypes. First, we analyzed and showed the genetic mutation distribution in the high- and low-risk score subgroups (Figure 8A,B). According to the cumulative incidence chart, the high-risk group had a higher incidence of somatic mutations than the low-risk group (94.32 vs. 90.53%). *TP53* (59%), *TTN* (39%), *FAT1* (20%), *CSMD3* (19%) and *SYNE1* (19%) were the top five genes with the highest mutation frequency in the low-risk group. Genes such as *TP53* (73%), *TTN* (34%), *FAT1* (23%), *CDKN2A* (18%), and *MUC16* (17%) had the top five mutation frequencies in the high-risk group. In general, oncogenes such as *MUC16* exhibited relatively low mutation rates in the high-risk group (18% vs. 17%), in contrast to anticancer genes such as *TP53*, which had comparatively high mutation rates in the high-risk group (59% vs. 73%). Subsequently, we analyzed the relationship between risk score and TMB score, and the results showed that there was a positive correlation between the risk score and TMB score (Supplementary Figure S1). Moreover, through redistribution of the TMB scores, the survival curve showed longer overall survival with low TMB levels (*p* = 0.01) (Figure 8C). Compared with the other groups, the low TMB and low-risk group had the best overall survival, while the high TMB and high-risk group had the worst prognosis (Figure 8D). Overall, it can be concluded that risk signatures may be associated with somatic mutations that affect tumor progression.

In order to explore the mutation status of 12 model genes in HNSC, we further analyzed them through the cBioPortal database. The results showed that the gene alteration types of *CDKN2A* were mainly deep deletion, mutation (putative driver), and the gene alteration types of EGFR were mainly amplification (Figure 8E). Based on the high mutation of *CDKN2A* in the risk model, we further demonstrated the type, site, and number of cases of *CDKN2A* gene modification (Figure 8F). We found 112 mutation sites between amino acids 0 and 156aa, of which 77 were truncating, 21 were missense, 11 were splice, two were inframe, and one was SV/Fusion. Among them, R80* was the most common mutation site (Figure 8G), and the *CDKN2A* mutation type of 23 patients was nonsense. In a word, these findings are expected to provide new insights into somatic variation in HNSC.

### 3.7. Predicting Responses to Small Drug Molecules

**Figure 9:**
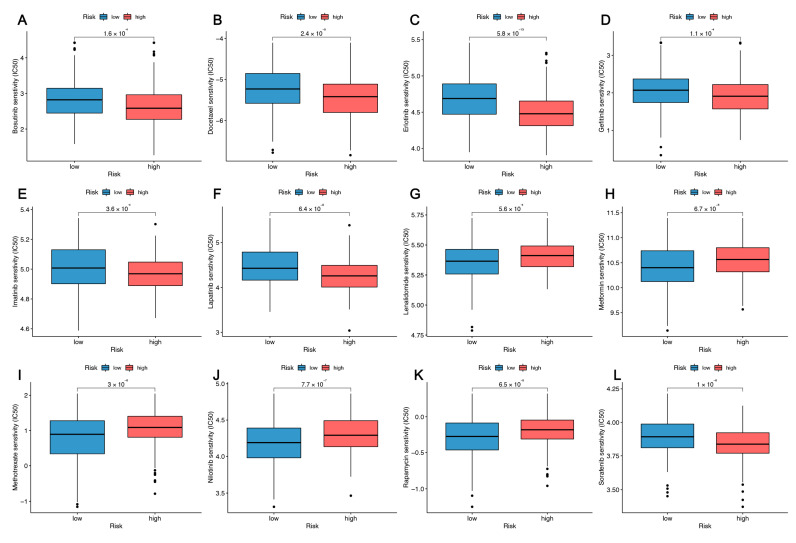
The prediction of the drug sensitivity in patients with head and neck squamous cell carcinoma. (**A**–**L**) The boxplot shows the mean difference in the estimated IC50 values for 12 representative drugs (bosutinib, docetaxel, erlotinib, gefitinib, imatinib, lapatinib, lenalidomide, metformin, methotrexate, nilotinib, rapamycin, sorafenib) between the two risk groups.

We further analyzed the differences in resistance potential between the two risk groups. The pRRophetic method was used to compare the estimated IC50 levels of the two groups of chemotherapeutics or inhibitors. Of these, 12 representative drugs are shown in Figure 9A–L. We found that the (lenalidomide, metformin, methotrexate, nilotinib, rapamycin) (*p* < 0.001) IC50 was significantly higher in the high-risk group than in the low-risk group, which means that patients in the high-risk group may not benefit from these drugs. Conversely, bosutinib, docetaxel, erlotinib, gefitinib, imatinib, lapatinib, sorafenib may be candidates for the treatment of patients in high-risk groups.

### 3.8. Gene Correlation Analysis

**Figure 10:**
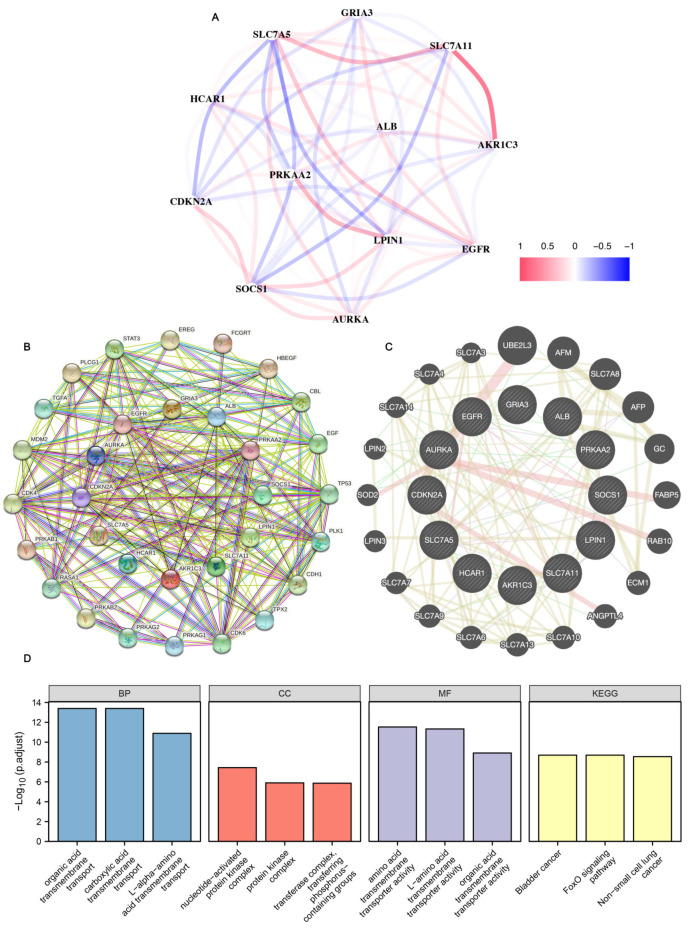
The model genes’ correlation analysis. (**A**) Construction of 12 gene-related correlation networks. (**B**) The model genes’ associated protein network mapped using STRING. (**C**) The model genes’ associated gene network mapped using GeneMANIA. (**D**) The functional enrichment analysis of the related genes and proteins.

In order to explore the correlation of the 12 genes, we analyzed the relationship between the 12 genes through the R language algorithm. As shown in Figure 10A, *SLC7A11* was significantly positively correlated with *SLC7A5* and *AKR1C3*; *SLC7A5* was significantly negatively correlated with *CDKN2A* and *LPIN1*. To investigate the model gene association networks, we used protein–protein, gene–gene interaction networks generated by STRING and GeneMANIA to show that 20 potential target proteins and 20 potential target genes interacted with the model gene (Figure 10B,C). Finally, based on protein and gene networks, we analyzed the functional enrichment and found that BP was enriched in organic acid transmembrane transport, carboxylic acid transmembrane transport, and L-alpha-amino acid transmembrane transport. CC is mainly enriched in nucleotide-activated protein kinase complex, the protein kinase complex, transferase complex, and transferring phosphorus-containing groups. MF was significantly enriched in amino acid transmembrane transporter activity, L-amino acid transmembrane transporter activity, and organic acid transmembrane transporter activity. KEGG analysis showed significant enrichment of many related pathways including bladder cancer, the FoxO signaling pathway, and non-small cell lung cancer (Figure 10D and Supplementary Table S3).

## 4. Discussion

Many current studies have focused on the effect of immune and ferroptosis-related gene expression on tumors [47,48,49,50,51]. The discovery of IFRGs could help to discover potential cancer targets. However, there is little information on the application of IFRGs in head and neck squamous cell carcinoma. Based on ImmPort, GeneCards, FerrDb, and the previous literature, we analyzed the expression profiles of IFRMs in humans [26,27] and screened out the differentially expressed IFRMs. The expression patterns of these IFRMs and the prognosis of each patient in the TCGA database were then evaluated, and 17 prognostic IFRMs were found. In addition, we established a new 12-mRNA prediction model. We divided head and neck squamous cell carcinoma into high-risk and low-risk types according to the risk scores obtained by this prediction model. The mechanism of action of this feature in head and neck squamous cell carcinoma was analyzed in more depth.

As a newly discovered mode of cell death, ferroptosis is mainly through lipid peroxidation and iron dependence. It has been reported that many immune-related indicators change during ferroptosis, so ferroptosis is closely related to the body’s immune microenvironment [47,52]. In order to explore the potential mechanism of the immune ferroptosis model in HNSC, we performed GSEA analysis and found that metabolic pathways such as hypoxia, glycolysis, cyclin, and EGFR signal transduction were enriched in high-risk groups. In low-risk groups, immunoregulatory proteins, NF-KB, PD1, MAPK, and other expression pathways were abundantly expressed. In addition, our KEGG and GO enrichment analysis showed that many immune and tumor-related pathways were enriched. Taken together, we could infer that immune ferroptosis and tumor-related pathways cross-talk with each other, leading to the development of tumors.

Previous studies have shown that ferroptosis is strongly related to tumor immune cell function, and some have said that it is an immunogenic cell death [53]. It has been found that CD8^+^T cells can induce ferroptosis in tumor cells [22]. Some studies have also shown that prostaglandin E2 (PGE2) aids in the immune escape of cancer [54,55]. We used ESTIMATE and CIBERSORT technology to count various tumor-aggressive immune cells through the TCGA database. The findings revealed that the high-risk group had considerably lower immunological, stromal, and ESTIMATE scores compared to the low-risk group. In addition, in the low-risk group, the expression levels of naive B cells, plasma cells, CD8^+^T cells, T follicular helper cells, Tregs, and gamma delta T cells were higher, while the expression levels of macrophages M0 and M2 and other immune cells in the high-risk group were also relatively high. The CD8^+^T cells, Treg cells, play a key role in tumor immunity [56]. We observed a significant decrease in CD8^+^T cells in high-risk populations, so we hypothesized that the effect of CD8^+^T cells would be relatively attenuated or slowed down in high-risk populations. Research has shown that immune checkpoint inhibition could improve the aggressiveness of the host immune system to tumor cells [57]. We found that the expression of specific immune checkpoint genes such as TNFSF9 and CD44 was significantly elevated in high-risk populations compared to low-risk populations. It might be possible to improve the prognosis of high-risk patients by enhancing the immune responsiveness.

The available clinical data suggested a relationship between genetic variation and immunotherapy response [58,59]. Risk score and mutation data showed significant differences at the transcriptional level between patients in the high-risk and low-risk groups. In this study, the variation in the *TTN* gene was significantly increased in the low-risk group, while variation in the TP53 gene was significantly increased in the high-risk group. In addition, we analyzed TMB as having a sensitivity to immunotherapy, and the results showed that the higher the incidence of TMB, the lower the subsequent survival rate. The following stratified survival curves revealed that the risk score had an independent prognostic prediction power of TMB, implying that TMB and the risk scores reflected all levels of immune biology. We also found from the cbioportal database that the model gene *CDKN2A* is highly mutated in HNSC, with deep deletion as the mutation type, which is common at the R80* site. It has been reported that the expression of the ferroptosis gene *CDKN2A* is related to the overall survival rate of human colorectal cancer [60]. In specific tumor subgroups of HNSCs, abnormalities in *TP53* and *CDKN2A* were highly correlated with higher TMB levels [61]. In certain C2 subtype cancers, *p16*^INK4A^ expression was markedly downregulated together with homozygous *CDKN2A* deletion [62]. In conclusion, there was some crosstalk between immune ferroptosis and tumor mutations, especially *CDKN2A*.

It is well-known that the survival of HNSC patients in high-risk and low-risk groups also differs due to their sensitivity to chemotherapy. Patients with head and neck squamous cell carcinoma generally survived longer after chemotherapy. For patients who were not sensitive to chemotherapy drugs, it was necessary to change the treatment strategy to improve the efficacy, but there was no relevant clinical information. We therefore analyzed the drug’s sensitivity and predicted its potential efficacy. A number of drugs might be considered therapeutic candidates for high-risk populations including bosutinib, docetaxel, erlotinib, gefitinib, imatinib, lapatinib, and sorafenib.

Finally, we tried to explore whether these 12 genes were intrinsically linked, and found that *SLC7A11* was significantly positively correlated with *SLC7A5* and *AKR1C3*; *SLC7A5* was significantly negatively correlated with *CDKN2A* and *LPIN1* through an algorithm analysis. We also constructed protein and gene networks. Interestingly, the GO analysis found a close relationship with transmembrane transport, and KEGG found that it was related to some tumor pathways. These were also prepared for the follow-up genetic mechanism research.

The work in this paper still needs to be further improved. The predictive role of the clinical cohort in its prognostic pattern had not been demonstrated, and we look forward to validating it with other cohorts in the next topic. Although we completed model gene correlation analysis and constructed an interaction network, molecular mechanism research still deserves attention. In conclusion, retrospective studies based on biological data still need further experimental and clinical data to confirm, and we look forward to further improvement after the epidemic is over.

## 5. Conclusions

In this experiment, only 12 IFRM prognosis prediction models were established, and this model had an independent prediction ability. The purpose of this study was to predict the survival state, immune microenvironment, and immunotherapy effect of HNSC patients, and provides a direction for new treatment strategies. Simultaneously, we examined the tumor mutation status in the risk model. Additionally, the relationship between model genes was discussed through the gene, protein network, and functional enrichment. A limitation of this study is that it has not been validated in an external cohort. We look forward to validation in other clinical cohorts after the popularization and improvement of medical data detection. All in all, this study was of great significance to explore the prognosis of patients with head and neck squamous cell carcinoma, and is helpful to its clinical application.

## Figures and Tables

**Table 1:** The GO and KEGG enrichment analysis.

Ontology	ID	Description	GeneRatio	BgRatio	*p*-Value	*p*. Adjust	q-Value
BP	GO:0006958	Complement activation, classical pathway	71/133	137/18,670	2.64 × 10^123^	3.96 × 10^120^	3.90 × 10^120^
BP	GO:0002455	Humoral immune response mediated by circulating immunoglobulin	71/133	150/18,670	1.75 × 10^119^	1.31 × 10^116^	1.29 × 10^116^
BP	GO:0006956	Complement activation	71/133	175/18,670	2.76 × 10^113^	1.38 × 10^110^	1.36 × 10^110^
BP	GO:0072376	Protein activation cascade	71/133	198/18,670	1.54 × 10^108^	5.77 × 10^106^	5.69 × 10^106^
BP	GO:0016064	Immunoglobulin mediated immune response	72/133	218/18,670	4.14 × 10^107^	1.24 × 10^104^	1.22 × 10^104^
CC	GO:0019814	Immunoglobulin complex	93/134	159/19,717	1.15 × 10^175^	1.27 × 10^173^	1.27 × 10^173^
CC	GO:0042571	Immunoglobulin complex, circulating	49/134	72/19,717	9.47 × 10^93^	5.26 × 10^91^	5.26 × 10^91^
CC	GO:0009897	External side of plasma membrane	54/134	393/19,717	1.09 × 10^56^	4.02 × 10^55^	4.02 × 10^55^
CC	GO:0072562	Blood microparticle	25/134	147/19,717	3.50 × 10^28^	9.72 × 10^27^	9.72 × 10^27^
MF	GO:0003823	Antigen binding	71/108	160/17,697	7.75 × 10^125^	1.15 × 10^122^	1.12 × 10^122^
MF	GO:0034987	Immunoglobulin receptor binding	48/108	76/17,697	9.71 × 10^92^	7.23 × 10^90^	7.00 × 10^90^
KEGG	hsa05340	Primary immunodeficiency	3/22	38/8076	1.39 × 10^04^	0.008	0.006
KEGG	hsa04064	NF-kappa B signaling pathway	4/22	104/8076	1.59 × 10^04^	0.008	0.006
KEGG	hsa04060	Cytokine–cytokine receptor interaction	5/22	295/8076	9.92 × 10^04^	0.035	0.025
KEGG	hsa01522	Endocrine resistance	3/22	98/8076	0.002	0.046	0.033
KEGG	hsa04061	Viral protein interaction with cytokine and cytokine receptor	3/22	100/8076	0.002	0.046	0.033

## Data Availability

The datasets generated during and/or analyzed during the current study are available from the corresponding author on reasonable request.

## Supplementary Materials

The following supporting information can be downloaded at: https://www.mdpi.com/article/10.3390/cancers14174099/s1, Supplementary Figure S1: Correlation between risk score and TMB score; Supplementary Table S1: List of differentially expressed genes in immune ferroptosis; Supplementary Table S2: Model genes and coefficients; Supplementary Table S3: Model-related gene enrichment analysis.
