# Supplementary figures and images for: Immunization Combined with Ferroptosis Related Genes to Construct a New Prognostic Model for Head and Neck Squamous Cell Carcinoma
Source: Cancers (Basel). 2022 Aug 24;14(17):4099. doi: 10.3390/cancers14174099 (PMC9454905; doi:10.3390/cancers14174099)

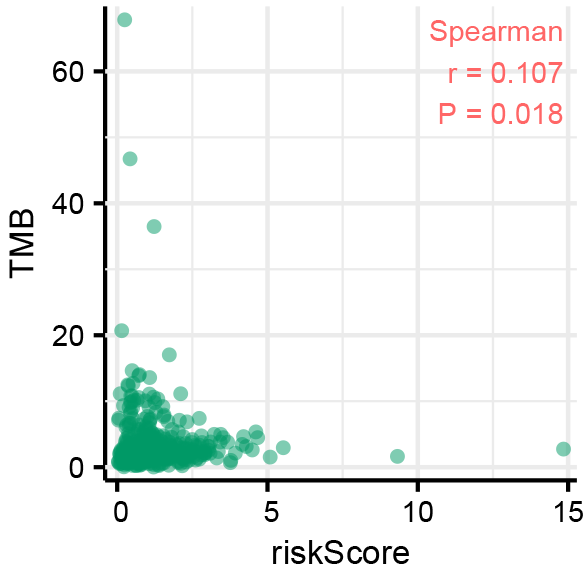

Supplement: Supplementary file 1 [file cancers-14-04099-s001.zip › Supplementary Figure S1.tif]
